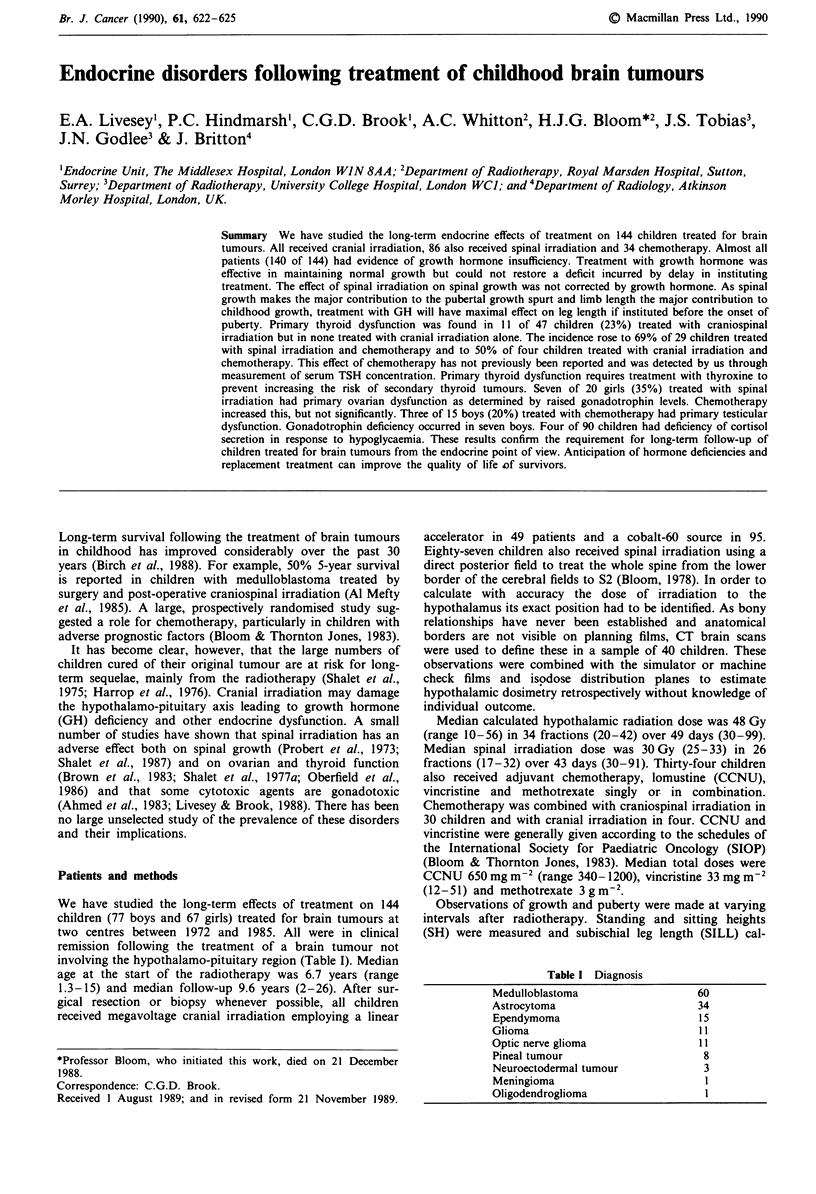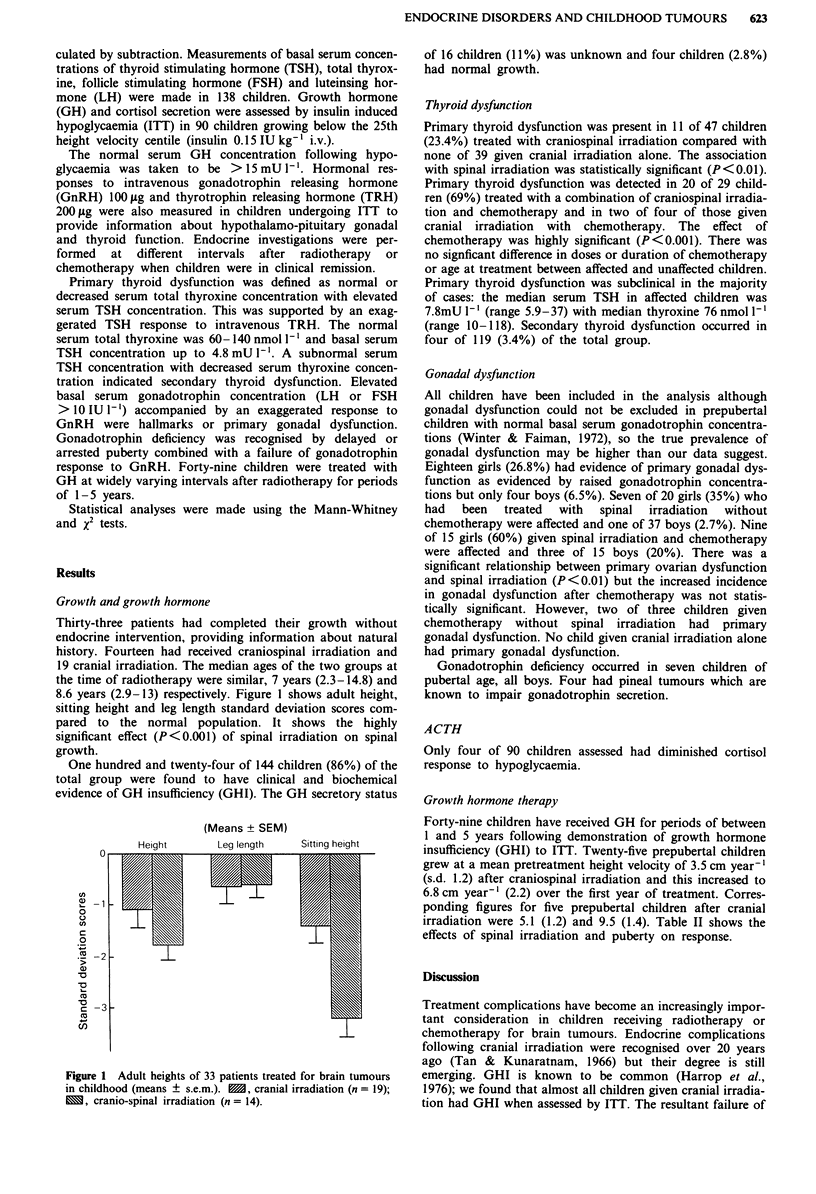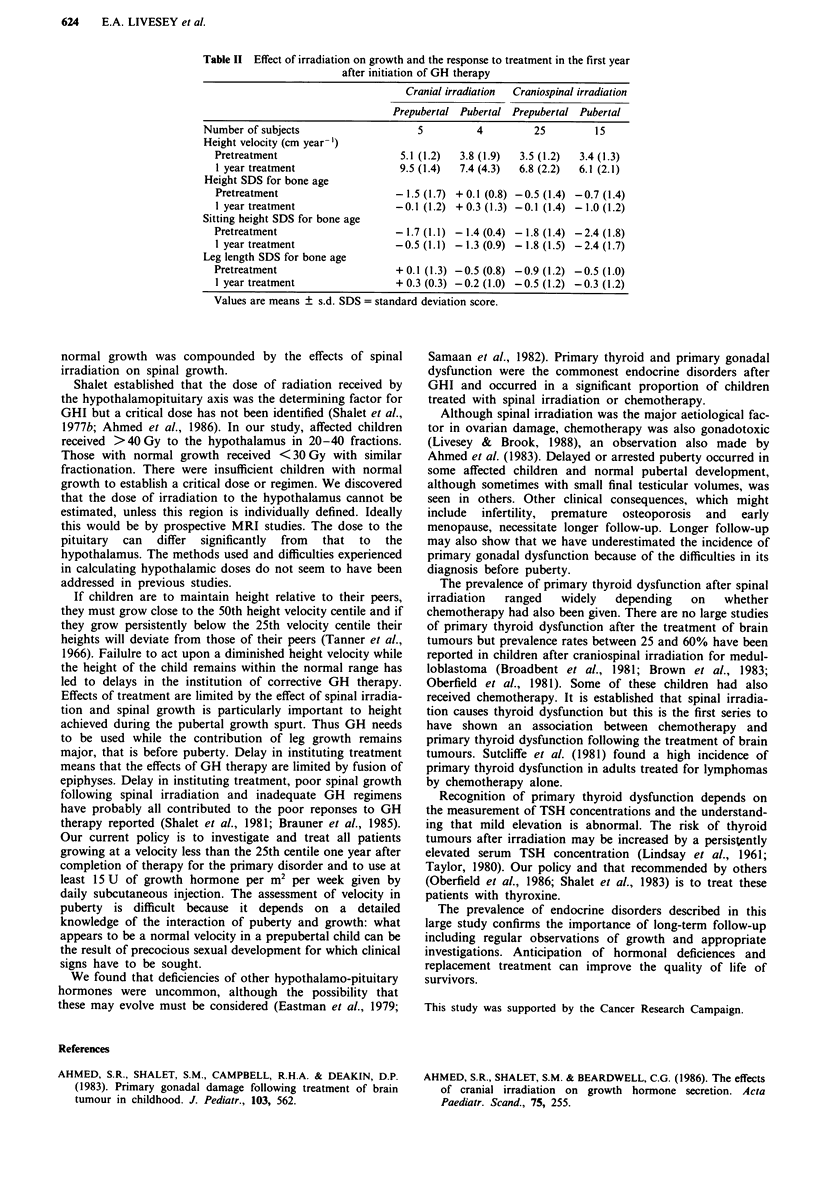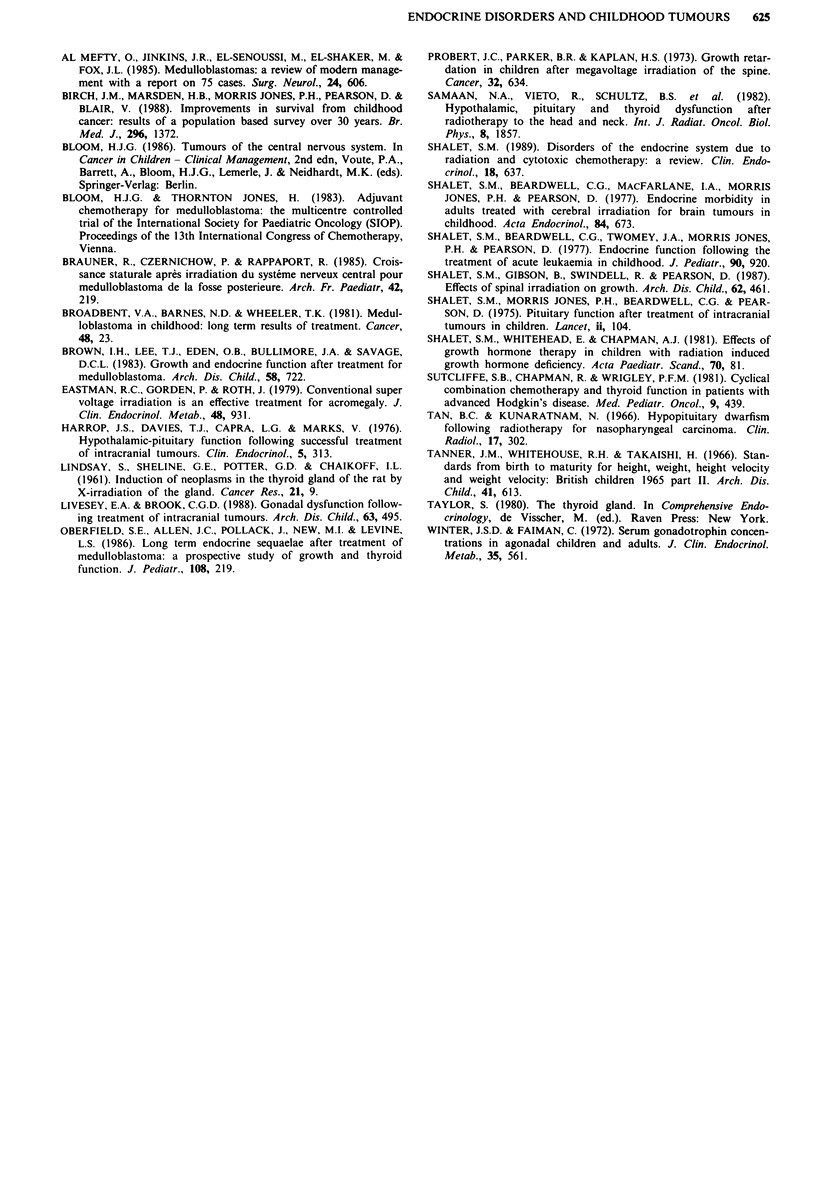# Endocrine disorders following treatment of childhood brain tumours.

**DOI:** 10.1038/bjc.1990.138

**Published:** 1990-04

**Authors:** E. A. Livesey, P. C. Hindmarsh, C. G. Brook, A. C. Whitton, H. J. Bloom, J. S. Tobias, J. N. Godlee, J. Britton

**Affiliations:** Endocrine Unit, Middlesex Hospital, London, UK.

## Abstract

We have studied the long-term endocrine effects of treatment on 144 children treated for brain tumours. All received cranial irradiation, 86 also received spinal irradiation and 34 chemotherapy. Almost all patients (140 of 144) had evidence of growth hormone insufficiency. Treatment with growth hormone was effective in maintaining normal growth but could not restore a deficit incurred by delay in instituting treatment. The effect of spinal irradiation on spinal growth was not corrected by growth hormone. As spinal growth makes the major contribution to the pubertal growth spurt and limb length the major contribution to childhood growth, treatment with GH will have maximal effect on leg length if instituted before the onset of puberty. Primary thyroid dysfunction was found in 11 of 47 children (23%) treated with craniospinal irradiation but in none treated with cranial irradiation alone. The incidence rose to 69% of 29 children treated with spinal irradiation and chemotherapy and to 50% of four children treated with cranial irradiation and chemotherapy. This effect of chemotherapy has not previously been reported and was detected by us through measurement of serum TSH concentration. Primary thyroid dysfunction requires treatment with thyroxine to prevent increasing the risk of secondary thyroid tumours. Seven of 20 girls (35%) treated with spinal irradiation had primary ovarian dysfunction as determined by raised gonadotrophin levels. Chemotherapy increased this, but not significantly. Three of 15 boys (20%) treated with chemotherapy had primary testicular dysfunction. Gonadotrophin deficiency occurred in seven boys. Four of 90 children had deficiency of cortisol secretion in response to hypoglycaemia. These results confirm the requirement for long-term follow-up of children treated for brain tumours from the endocrine point of view. Anticipation of hormone deficiencies and replacement treatment can improve the quality of life of survivors.


Br. J. Cancer (1990), 61, 622-625 © Macmillan Press Ltd., 1990

### Endocrine disorders following treatment of childhood brain tumours

E.A. Livesey[1], P.C. Hindmarsh[1], C.G.D. Brook[1], A.C. Whitton[2], H.J.G. Bloom*[2], J.S. Tobias[3], J.N. Godlee[3] & J. Britton[4]

[1]*Endocrine Unit, The Middlesex Hospital, London W1N 8AA;* [2]*Department of Radiotherapy, Royal Marsden Hospital, Sutton, Surrey;* [3]*Department of Radiotherapy, University College Hospital, London WC1; and* [4]*Department of Radiology, Atkinson Morley Hospital, London, UK.*

**Summary** We have studied the long-term endocrine effects of treatment on 144 children treated for brain tumours. All received cranial irradiation, 86 also received spinal irradiation and 34 chemotherapy. Almost all patients (140 of 144) had evidence of growth hormone insufficiency. Treatment with growth hormone was effective in maintaining normal growth but could not restore a deficit incurred by delay in instituting treatment. The effect of spinal irradiation on spinal growth was not corrected by growth hormone. As spinal growth makes the major contribution to the pubertal growth spurt and limb length the major contribution to childhood growth, treatment with GH will have maximal effect on leg length if instituted before the onset of puberty. Primary thyroid dysfunction was found in 11 of 47 children (23%) treated with craniospinal irradiation but in none treated with cranial irradiation alone. The incidence rose to 69% of 29 children treated with spinal irradiation and chemotherapy and to 50% of four children treated with cranial irradiation and chemotherapy. This effect of chemotherapy has not previously been reported and was detected by us through measurement of serum TSH concentration. Primary thyroid dysfunction requires treatment with thyroxine to prevent increasing the risk of secondary thyroid tumours. Seven of 20 girls (35%) treated with spinal irradiation had primary ovarian dysfunction as determined by raised gonadotrophin levels. Chemotherapy increased this, but not significantly. Three of 15 boys (20%) treated with chemotherapy had primary testicular dysfunction. Gonadotrophin deficiency occurred in seven boys. Four of 90 children had deficiency of cortisol secretion in response to hypoglycaemia. These results confirm the requirement for long-term follow-up of children treated for brain tumours from the endocrine point of view. Anticipation of hormone deficiencies and replacement treatment can improve the quality of life of survivors.

Long-term survival following the treatment of brain tumours in childhood has improved considerably over the past 30 years (Birch *et al.*, 1988). For example, 50% 5-year survival is reported in children with medulloblastoma treated by surgery and post-operative craniospinal irradiation (Al Mefty *et al.*, 1985). A large, prospectively randomised study suggested a role for chemotherapy, particularly in children with adverse prognostic factors (Bloom & Thornton Jones, 1983).

It has become clear, however, that the large numbers of children cured of their original tumour are at risk for long-term sequelae, mainly from the radiotherapy (Shalet *et al.*, 1975; Harrop *et al.*, 1976). Cranial irradiation may damage the hypothalamo-pituitary axis leading to growth hormone (GH) deficiency and other endocrine dysfunction. A small number of studies have shown that spinal irradiation has an adverse effect both on spinal growth (Probert *et al.*, 1973; Shalet *et al.*, 1987) and on ovarian and thyroid function (Brown *et al.*, 1983; Shalet *et al.*, 1977*a*; Oberfield *et al.*, 1986) and that some cytotoxic agents are gonadotoxic (Ahmed *et al.*, 1983; Livesey & Brook, 1988). There has been no large unselected study of the prevalence of these disorders and their implications.

### Patients and methods

We have studied the long-term effects of treatment on 144 children (77 boys and 67 girls) treated for brain tumours at two centres between 1972 and 1985. All were in clinical remission following the treatment of a brain tumour not involving the hypothalamo-pituitary region (Table I). Median age at the start of the radiotherapy was 6.7 years (range 1.3-15) and median follow-up 9.6 years (2-26). After surgical resection or biopsy whenever possible, all children received megavoltage cranial irradiation employing a linear accelerator in 49 patients and a cobalt-60 source in 95. Eighty-seven children also received spinal irradiation using a direct posterior field to treat the whole spine from the lower border of the cerebral fields to S2 (Bloom, 1978). In order to calculate with accuracy the dose of irradiation to the hypothalamus its exact position had to be identified. As bony relationships have never been established and anatomical borders are not visible on planning films, CT brain scans were used to define these in a sample of 40 children. These observations were combined with the simulator or machine check films and isodose distribution planes to estimate hypothalamic dosimetry retrospectively without knowledge of individual outcome.

Median calculated hypothalamic radiation dose was 48 Gy (range 10-56) in 34 fractions (20-42) over 49 days (30-99). Median spinal irradiation dose was 30 Gy (25-33) in 26 fractions (17-32) over 43 days (30-91). Thirty-four children also received adjuvant chemotherapy, lomustine (CCNU), vincristine and methotrexate singly or in combination. Chemotherapy was combined with craniospinal irradiation in 30 children and with cranial irradiation in four. CCNU and vincristine were generally given according to the schedules of the International Society for Paediatric Oncology (SIOP) (Bloom & Thornton Jones, 1983). Median total doses were CCNU 650 mg $m^{-2}$ (range 340-1200), vincristine 33 mg $m^{-2}$ (12-51) and methotrexate 3 g $m^{-2}$.

Observations of growth and puberty were made at varying intervals after radiotherapy. Standing and sitting heights (SH) were measured and subischial leg length (SILL) cal-

**Table I** Diagnosis

| | |
|---|---|
| Medulloblastoma | 60 |
| Astrocytoma | 34 |
| Ependymoma | 15 |
| Glioma | 11 |
| Optic nerve glioma | 11 |
| Pineal tumour | 8 |
| Neuroectodermal tumour | 3 |
| Meningioma | 1 |
| Oligodendroglioma | 1 |

*Professor Bloom, who initiated this work, died on 21 December 1988.

Correspondence: C.G.D. Brook.

Received 1 August 1989; and in revised form 21 November 1989.

culated by subtraction. Measurements of basal serum concentrations of thyroid stimulating hormone (TSH), total thyroxine, follicle stimulating hormone (FSH) and luteinsing hormone (LH) were made in 138 children. Growth hormone (GH) and cortisol secretion were assessed by insulin induced hypoglycaemia (ITT) in 90 children growing below the 25th height velocity centile (insulin 0.15 IU $kg^{-1}$ i.v.).

The normal serum GH concentration following hypoglycaemia was taken to be $>15\,mU\,l^{-1}$. Hormonal responses to intravenous gonadotrophin releasing hormone (GnRH) 100 μg and thyrotrophin releasing hormone (TRH) 200 μg were also measured in children undergoing ITT to provide information about hypothalamo-pituitary gonadal and thyroid function. Endocrine investigations were performed at different intervals after radiotherapy or chemotherapy when children were in clinical remission.

Primary thyroid dysfunction was defined as normal or decreased serum total thyroxine concentration with elevated serum TSH concentration. This was supported by an exaggerated TSH response to intravenous TRH. The normal serum total thyroxine was 60–140 nmol $l^{-1}$ and basal serum TSH concentration up to 4.8 mU $l^{-1}$. A subnormal serum TSH concentration with decreased serum thyroxine concentration indicated secondary thyroid dysfunction. Elevated basal serum gonadotrophin concentration (LH or FSH $>10\,IU\,l^{-1}$) accompanied by an exaggerated response to GnRH were hallmarks or primary gonadal dysfunction. Gonadotrophin deficiency was recognised by delayed or arrested puberty combined with a failure of gonadotrophin response to GnRH. Forty-nine children were treated with GH at widely varying intervals after radiotherapy for periods of 1–5 years.

Statistical analyses were made using the Mann-Whitney and $\chi^2$ tests.

### Results

### *Growth and growth hormone*

Thirty-three patients had completed their growth without endocrine intervention, providing information about natural history. Fourteen had received craniospinal irradiation and 19 cranial irradiation. The median ages of the two groups at the time of radiotherapy were similar, 7 years (2.3–14.8) and 8.6 years (2.9–13) respectively. Figure 1 shows adult height, sitting height and leg length standard deviation scores compared to the normal population. It shows the highly significant effect ($P<0.001$) of spinal irradiation on spinal growth.

One hundred and twenty-four of 144 children (86%) of the total group were found to have clinical and biochemical evidence of GH insufficiency (GHI). The GH secretory status of 16 children (11%) was unknown and four children (2.8%) had normal growth.

**Figure 1** Adult heights of 33 patients treated for brain tumours in childhood (means ± s.e.m.). ▨, cranial irradiation ($n$ = 19); ▧, cranio-spinal irradiation ($n$ = 14).

### *Thyroid dysfunction*

Primary thyroid dysfunction was present in 11 of 47 children (23.4%) treated with craniospinal irradiation compared with none of 39 given cranial irradiation alone. The association with spinal irradiation was statistically significant ($P<0.01$). Primary thyroid dysfunction was detected in 20 of 29 children (69%) treated with a combination of craniospinal irradiation and chemotherapy and in two of four of those given cranial irradiation with chemotherapy. The effect of chemotherapy was highly significant ($P<0.001$). There was no signficant difference in doses or duration of chemotherapy or age at treatment between affected and unaffected children. Primary thyroid dysfunction was subclinical in the majority of cases: the median serum TSH in affected children was 7.8mU $l^{-1}$ (range 5.9–37) with median thyroxine 76 nmol $l^{-1}$ (range 10–118). Secondary thyroid dysfunction occurred in four of 119 (3.4%) of the total group.

### *Gonadal dysfunction*

All children have been included in the analysis although gonadal dysfunction could not be excluded in prepubertal children with normal basal serum gonadotrophin concentrations (Winter & Faiman, 1972), so the true prevalence of gonadal dysfunction may be higher than our data suggest. Eighteen girls (26.8%) had evidence of primary gonadal dysfunction as evidenced by raised gonadotrophin concentrations but only four boys (6.5%). Seven of 20 girls (35%) who had been treated with spinal irradiation without chemotherapy were affected and one of 37 boys (2.7%). Nine of 15 girls (60%) given spinal irradiation and chemotherapy were affected and three of 15 boys (20%). There was a significant relationship between primary ovarian dysfunction and spinal irradiation ($P<0.01$) but the increased incidence in gonadal dysfunction after chemotherapy was not statistically significant. However, two of three children given chemotherapy without spinal irradiation had primary gonadal dysfunction. No child given cranial irradiation alone had primary gonadal dysfunction.

Gonadotrophin deficiency occurred in seven children of pubertal age, all boys. Four had pineal tumours which are known to impair gonadotrophin secretion.

### *ACTH*

Only four of 90 children assessed had diminished cortisol response to hypoglycaemia.

### *Growth hormone therapy*

Forty-nine children have received GH for periods of between 1 and 5 years following demonstration of growth hormone insufficiency (GHI) to ITT. Twenty-five prepubertal children grew at a mean pretreatment height velocity of 3.5 cm $year^{-1}$ (s.d. 1.2) after craniospinal irradiation and this increased to 6.8 cm $year^{-1}$ (2.2) over the first year of treatment. Corresponding figures for five prepubertal children after cranial irradiation were 5.1 (1.2) and 9.5 (1.4). Table II shows the effects of spinal irradiation and puberty on response.

### Discussion

Treatment complications have become an increasingly important consideration in children receiving radiotherapy or chemotherapy for brain tumours. Endocrine complications following cranial irradiation were recognised over 20 years ago (Tan & Kunaratnam, 1966) but their degree is still emerging. GHI is known to be common (Harrop *et al.*, 1976); we found that almost all children given cranial irradiation had GHI when assessed by ITT. The resultant failure of

**Table II** Effect of irradiation on growth and the response to treatment in the first year after initiation of GH therapy

| | *Cranial irradiation* | | *Craniospinal irradiation* | |
|---|---|---|---|---|
| | *Prepubertal* | *Pubertal* | *Prepubertal* | *Pubertal* |
| Number of subjects | 5 | 4 | 25 | 15 |
| Height velocity (cm $year^{-1}$) | | | | |
| Pretreatment | 5.1 (1.2) | 3.8 (1.9) | 3.5 (1.2) | 3.4 (1.3) |
| 1 year treatment | 9.5 (1.4) | 7.4 (4.3) | 6.8 (2.2) | 6.1 (2.1) |
| Height SDS for bone age | | | | |
| Pretreatment | −1.5 (1.7) | +0.1 (0.8) | −0.5 (1.4) | −0.7 (1.4) |
| 1 year treatment | −0.1 (1.2) | +0.3 (1.3) | −0.1 (1.4) | −1.0 (1.2) |
| Sitting height SDS for bone age | | | | |
| Pretreatment | −1.7 (1.1) | −1.4 (0.4) | −1.8 (1.4) | −2.4 (1.8) |
| 1 year treatment | −0.5 (1.1) | −1.3 (0.9) | −1.8 (1.5) | −2.4 (1.7) |
| Leg length SDS for bone age | | | | |
| Pretreatment | +0.1 (1.3) | −0.5 (0.8) | −0.9 (1.2) | −0.5 (1.0) |
| 1 year treatment | +0.3 (0.3) | −0.2 (1.0) | −0.5 (1.2) | −0.3 (1.2) |

Values are means ± s.d. SDS = standard deviation score.

normal growth was compounded by the effects of spinal irradiation on spinal growth.

Shalet established that the dose of radiation received by the hypothalamopituitary axis was the determining factor for GHI but a critical dose has not been identified (Shalet *et al.*, 1977*b*; Ahmed *et al.*, 1986). In our study, affected children received >40 Gy to the hypothalamus in 20–40 fractions. Those with normal growth received <30 Gy with similar fractionation. There were insufficient children with normal growth to establish a critical dose or regimen. We discovered that the dose of irradiation to the hypothalamus cannot be estimated, unless this region is individually defined. Ideally this would be by prospective MRI studies. The dose to the pituitary can differ significantly from that to the hypothalamus. The methods used and difficulties experienced in calculating hypothalamic doses do not seem to have been addressed in previous studies.

If children are to maintain height relative to their peers, they must grow close to the 50th height velocity centile and if they grow persistently below the 25th velocity centile their heights will deviate from those of their peers (Tanner *et al.*, 1966). Failulre to act upon a diminished height velocity while the height of the child remains within the normal range has led to delays in the institution of corrective GH therapy. Effects of treatment are limited by the effect of spinal irradiation and spinal growth is particularly important to height achieved during the pubertal growth spurt. Thus GH needs to be used while the contribution of leg growth remains major, that is before puberty. Delay in instituting treatment means that the effects of GH therapy are limited by fusion of epiphyses. Delay in instituting treatment, poor spinal growth following spinal irradiation and inadequate GH regimens have probably all contributed to the poor reponses to GH therapy reported (Shalet *et al.*, 1981; Brauner *et al.*, 1985). Our current policy is to investigate and treat all patients growing at a velocity less than the 25th centile one year after completion of therapy for the primary disorder and to use at least 15 U of growth hormone per $m^2$ per week given by daily subcutaneous injection. The assessment of velocity in puberty is difficult because it depends on a detailed knowledge of the interaction of puberty and growth: what appears to be a normal velocity in a prepubertal child can be the result of precocious sexual development for which clinical signs have to be sought.

We found that deficiencies of other hypothalamo-pituitary hormones were uncommon, although the possibility that these may evolve must be considered (Eastman *et al.*, 1979; Samaan *et al.*, 1982). Primary thyroid and primary gonadal dysfunction were the commonest endocrine disorders after GHI and occurred in a significant proportion of children treated with spinal irradiation or chemotherapy.

Although spinal irradiation was the major aetiological factor in ovarian damage, chemotherapy was also gonadotoxic (Livesey & Brook, 1988), an observation also made by Ahmed *et al.* (1983). Delayed or arrested puberty occurred in some affected children and normal pubertal development, although sometimes with small final testicular volumes, was seen in others. Other clinical consequences, which might include infertility, premature osteoporosis and early menopause, necessitate longer follow-up. Longer follow-up may also show that we have underestimated the incidence of primary gonadal dysfunction because of the difficulties in its diagnosis before puberty.

The prevalence of primary thyroid dysfunction after spinal irradiation ranged widely depending on whether chemotherapy had also been given. There are no large studies of primary thyroid dysfunction after the treatment of brain tumours but prevalence rates between 25 and 60% have been reported in children after craniospinal irradiation for medulloblastoma (Broadbent *et al.*, 1981; Brown *et al.*, 1983; Oberfield *et al.*, 1981). Some of these children had also received chemotherapy. It is established that spinal irradiation causes thyroid dysfunction but this is the first series to have shown an association between chemotherapy and primary thyroid dysfunction following the treatment of brain tumours. Sutcliffe *et al.* (1981) found a high incidence of primary thyroid dysfunction in adults treated for lymphomas by chemotherapy alone.

Recognition of primary thyroid dysfunction depends on the measurement of TSH concentrations and the understanding that mild elevation is abnormal. The risk of thyroid tumours after irradiation may be increased by a persistently elevated serum TSH concentration (Lindsay *et al.*, 1961; Taylor, 1980). Our policy and that recommended by others (Oberfield *et al.*, 1986; Shalet *et al.*, 1983) is to treat these patients with thyroxine.

The prevalence of endocrine disorders described in this large study confirms the importance of long-term follow-up including regular observations of growth and appropriate investigations. Anticipation of hormonal deficiences and replacement treatment can improve the quality of life of survivors.

This study was supported by the Cancer Research Campaign.

**References**

AHMED, S.R., SHALET, S.M., CAMPBELL, R.H.A. & DEAKIN, D.P. (1983). Primary gonadal damage following treatment of brain tumour in childhood. *J. Pediatr.*, **103**, 562.

AHMED, S.R., SHALET, S.M. & BEARDWELL, C.G. (1986). The effects of cranial irradiation on growth hormone secretion. *Acta Paediatr. Scand.*, **75**, 255.

AL MEFTY, O., JINKINS, J.R., EL-SENOUSSI, M., EL-SHAKER, M. & FOX, J.L. (1985). Medulloblastomas: a review of modern management with a report on 75 cases. *Surg. Neurol.*, **24**, 606.

BIRCH, J.M., MARSDEN, H.B., MORRIS JONES, P.H., PEARSON, D. & BLAIR, V. (1988). Improvements in survival from childhood cancer: results of a population based survey over 30 years. *Br. Med. J.*, **296**, 1372.

BLOOM, H.J.G. (1986). Tumours of the central nervous system. In *Cancer in Children – Clinical Management*, 2nd edn, Voute, P.A., Barrett, A., Bloom, H.J.G., Lemerle, J. & Neidhardt, M.K. (eds). Springer-Verlag: Berlin.

BLOOM, H.J.G. & THORNTON JONES, H. (1983). Adjuvant chemotherapy for medulloblastoma: the multicentre controlled trial of the International Society for Paediatric Oncology (SIOP). Proceedings of the 13th International Congress of Chemotherapy, Vienna.

BRAUNER, R., CZERNICHOW, P. & RAPPAPORT, R. (1985). Croissance staturale après irradiation du systéme nerveux central pour medulloblastoma de la fosse posterieure. *Arch. Fr. Paediatr*, **42**, 219.

BROADBENT, V.A., BARNES, N.D. & WHEELER, T.K. (1981). Medulloblastoma in childhood: long term results of treatment. *Cancer*, **48**, 23.

BROWN, I.H., LEE, T.J., EDEN, O.B., BULLIMORE, J.A. & SAVAGE, D.C.L. (1983). Growth and endocrine function after treatment for medulloblastoma. *Arch. Dis. Child.*, **58**, 722.

EASTMAN, R.C., GORDEN, P. & ROTH, J. (1979). Conventional super voltage irradiation is an effective treatment for acromegaly. *J. Clin. Endocrinol. Metab.*, **48**, 931.

HARROP, J.S., DAVIES, T.J., CAPRA, L.G. & MARKS, V. (1976). Hypothalamic-pituitary function following successful treatment of intracranial tumours. *Clin. Endocrinol.*, **5**, 313.

LINDSAY, S., SHELINE, G.E., POTTER, G.D. & CHAIKOFF, I.L. (1961). Induction of neoplasms in the thyroid gland of the rat by X-irradiation of the gland. *Cancer Res.*, **21**, 9.

LIVESEY, E.A. & BROOK, C.G.D. (1988). Gonadal dysfunction following treatment of intracranial tumours. *Arch. Dis. Child.*, **63**, 495.

OBERFIELD, S.E., ALLEN, J.C., POLLACK, J., NEW, M.I. & LEVINE, L.S. (1986). Long term endocrine sequaelae after treatment of medulloblastoma: a prospective study of growth and thyroid function. *J. Pediatr.*, **108**, 219.

PROBERT, J.C., PARKER, B.R. & KAPLAN, H.S. (1973). Growth retardation in children after megavoltage irradiation of the spine. *Cancer*, **32**, 634.

SAMAAN, N.A., VIETO, R., SCHULTZ, B.S. *et al.* (1982). Hypothalamic, pituitary and thyroid dysfunction after radiotherapy to the head and neck. *Int. J. Radiat. Oncol. Biol. Phys.*, **8**, 1857.

SHALET, S.M. (1989). Disorders of the endocrine system due to radiation and cytotoxic chemotherapy: a review. *Clin. Endocrinol.*, **18**, 637.

SHALET, S.M., BEARDWELL, C.G., MACFARLANE, I.A., MORRIS JONES, P.H. & PEARSON, D. (1977). Endocrine morbidity in adults treated with cerebral irradiation for brain tumours in childhood. *Acta Endocrinol.*, **84**, 673.

SHALET, S.M., BEARDWELL, C.G., TWOMEY, J.A., MORRIS JONES, P.H. & PEARSON, D. (1977). Endocrine function following the treatment of acute leukaemia in childhood. *J. Pediatr.*, **90**, 920.

SHALET, S.M., GIBSON, B., SWINDELL, R. & PEARSON, D. (1987). Effects of spinal irradiation on growth. *Arch. Dis. Child.*, **62**, 461.

SHALET, S.M., MORRIS JONES, P.H., BEARDWELL, C.G. & PEARSON, D. (1975). Pituitary function after treatment of intracranial tumours in children. *Lancet*, **ii**, 104.

SHALET, S.M., WHITEHEAD, E. & CHAPMAN, A.J. (1981). Effects of growth hormone therapy in children with radiation induced growth hormone deficiency. *Acta Paediatr. Scand.*, **70**, 81.

SUTCLIFFE, S.B., CHAPMAN, R. & WRIGLEY, P.F.M. (1981). Cyclical combination chemotherapy and thyroid function in patients with advanced Hodgkin's disease. *Med. Pediatr. Oncol.*, **9**, 439.

TAN, B.C. & KUNARATNAM, N. (1966). Hypopituitary dwarfism following radiotherapy for nasopharyngeal carcinoma. *Clin. Radiol.*, **17**, 302.

TANNER, J.M., WHITEHOUSE, R.H. & TAKAISHI, H. (1966). Standards from birth to maturity for height, weight, height velocity and weight velocity: British children 1965 part II. *Arch. Dis. Child.*, **41**, 613.

TAYLOR, S. (1980). The thyroid gland. In *Comprehensive Endocrinology*, de Visscher, M. (ed.). Raven Press: New York.

WINTER, J.S.D. & FAIMAN, C. (1972). Serum gonadotrophin concentrations in agonadal children and adults. *J. Clin. Endocrinol. Metab.*, **35**, 561.